# P-529. Oseltamivir Drug-Drug Interactions and Risk of Neuropsychiatric Events

**DOI:** 10.1093/ofid/ofaf695.744

**Published:** 2026-01-11

**Authors:** James W Antoon, Derek Williams, Avirath Vaidya, Mert Sekmen, Yuwei Zhu, Carlos G Grijalva

**Affiliations:** Vanderbilt University Medical Center, Nashville, TN; Vanderbilt University Medical Center, Nashville, TN; Vanderbilt University Medical Center, Nashville, TN; Vanderbilt University Medical Center, Nashville, TN; VUMC, NASHVILLE, Tennessee; Vanderbilt University Medical Center, Nashville, TN

## Abstract

**Background:**

Reports of neuropsychiatric events related to oseltamivir use have led to public health concerns. Oseltamivir is transported out of the CSF by the P-glycoprotein (P-gp) system. We explored whether concurrent use of oseltamivir and P-gp inhibitors, which would increase CSF concentrations of oseltamivir, is associated with neuropsychiatric events.

**Figure:**
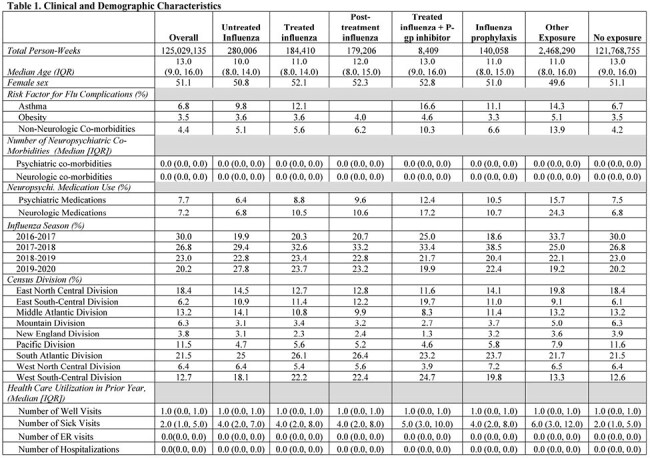

Notes:

1.Person-time accrued for each subject beginning on the first day of the influenza season and continuing through the earliest occurrence of an incident neuropsychiatric outcome event (see definition below), loss of enrollment, death, age 18 years, or end of the study.

2. Characterization of study exposures and covariates was conducted throughout follow-up for all individuals, began at cohort entry and was measured at the person-day level, allowing exposures and covariates to be time-dependent. Importantly, the start date for all influenza episodes (both treated and untreated) began on the same date, i.e., the date of influenza diagnosis.

3. Other exposure includes other influenza antivirals other than oseltamivir (e.g. baloxavir, zanamivir, peramivir) alone or in combination with influenza, oseltamivir + P-gp modifiers without influenza exposure and P-gp modifiers alone.

4. Outcome definition: The outcome definition includes both neurologic (seizures, encephalitis, altered mental status, ataxia/movement disorders, vision changes, dizziness, headache, sleeping disorders) and psychiatric (suicidal or self-harm behaviors, mood disorders, psychosis/hallucination) events

**Figure 1.**
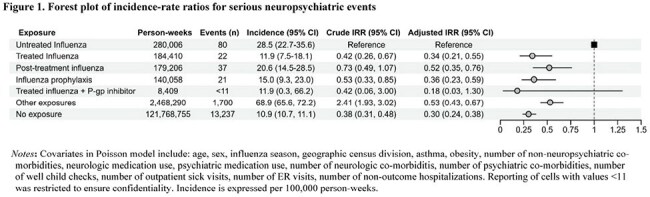
Forest plot of incidence-rate ratios for serious neuropsychiatric events

**Methods:**

We assembled a retrospective cohort of children 5-17 years and followed them during the 2016-2020 influenza seasons in the Marketscan database. Each person-day of follow-up was assigned to one of six cohorts: 1) untreated influenza (10 day period after influenza diagnosis), 2) treated influenza (influenza + oseltamivir dispensing plus days supply) 3) post-treatment influenza (period between completion of oseltamivir and 10 days after influenza diagnosis), 4) treated influenza+P-gp inhibitor, 5) other (other antivirals or P-gp modifiers) or 6) no exposure. The outcome was serious neuropsychiatric events resulting in hospitalization and identified using a validated algorithm (PPV ∼90%) (Table 1). Incidence-rate ratios (IRRs) with 95% CI were estimated using Poisson regression model adjusting for relevant confounders. Sensitivity analyses were performed including alternative exposure and outcome definitions as well as unmeasured confounding.

**Figure:**
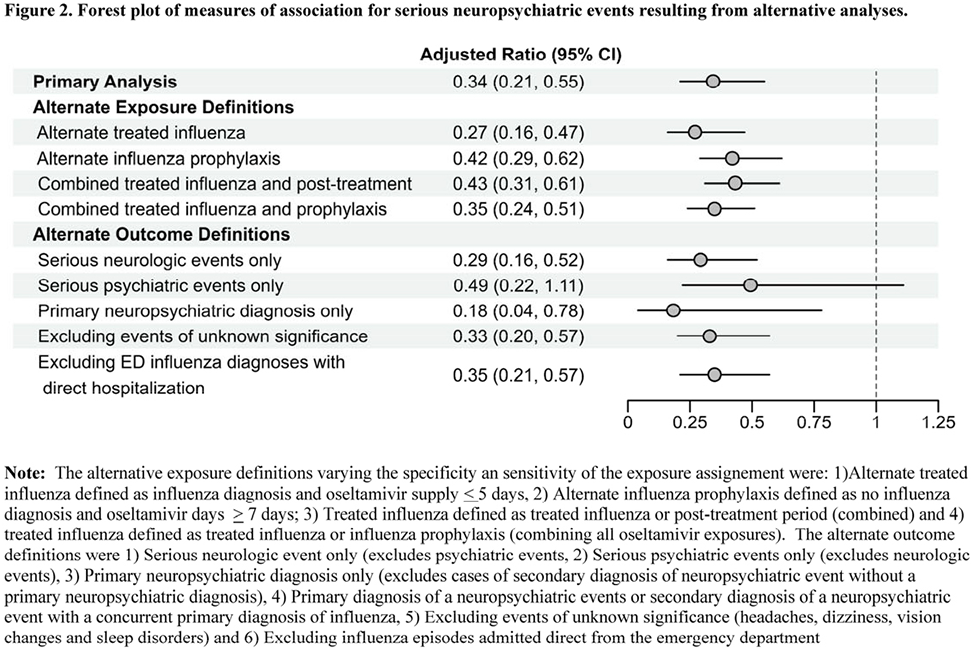

**Results:**

A total of 5,481,906 children contributed 125,029,135 person-weeks of follow-up, encompassing 400,031 person-weeks of influenza and 15,098 neuropsychiatric events. Median age was 13.0 (IQR 9, 16) years (Table 1). Compared with untreated influenza, episodes of influenza treated with oseltamivir had lower risk of neuropsychiatric events (treated influenza IRR 0.34, 95% CI 0.21-0.55; post-treatment influenza IRR 0.52, 95% CI 0.35-0.76; treated influenza+P-gp inhibitor 0.18, 95% CI 0.0.3-1.30) (Figure 1). Alternate analyses suggest misclassification (Figure 2) or unmeasured confounding (measured E-value 5.33) would not explain the findings.

**Conclusion:**

Oseltamivir use was associated with a reduced risk of serious pediatric neuropsychiatric events, even when used concurrently with medications that would theoretically increase CSF concentrations of the drug. These findings provide reassurance to patients and providers as to the safety of oseltamivir use.

**Disclosures:**

James W. Antoon, MD, PhD, MPH, AstraZeneca: Advisor/Consultant|NIH: Grant/Research Support Carlos G. Grijalva, MD MPH, AHRQ: Grant/Research Support|CDC: Grant/Research Support|GSK: Advisor/Consultant|Merck: Advisor/Consultant|NIH: Grant/Research Support|Syneos Health: Grant/Research Support

